# Case Report: A rare case of MET-amplified gastric cancer with systemic metastasis: remarkable efficacy of crizotinib and the role of precision medicine

**DOI:** 10.3389/fonc.2025.1555801

**Published:** 2025-08-08

**Authors:** Yan Shen, Yaxin Xu, Jing Sun

**Affiliations:** ^1^ Department of Oncology, The First Affiliated Hospital with Nanjing Medical University, Nanjing, China; ^2^ The First School of Clinical Medicine, Nanjing Medical University, Nanjing, China

**Keywords:** targeted therapy, MET-amplified, precision medicine, crizotinib, gastric cancer

## Abstract

Gastric cancer remains one of the most prevalent gastrointestinal malignancies, with certain subtypes, such as poorly cohesive carcinoma—including signet ring cell carcinoma (SRCC)—exhibiting aggressive progression and poor prognosis. Mesenchymal epithelial transition (MET) amplification, a relatively rare oncogenic driver in gastric cancer (~2–10.2% of cases), has been associated with resistance to conventional therapies and dismal survival (median <6 months in metastatic cases). While MET inhibitors such as crizotinib have shown efficacy in MET-altered non-small cell lung cancer (NSCLC), their role in gastric cancer remains uncertain due to tumor heterogeneity and the lack of robust clinical evidence. We report a case of a female patient with MET-amplified metastatic gastric cancer and systemic bone marrow involvement. Despite eventual disease progression, the initial response to crizotinib was remarkable, with rapid hematologic recovery (platelets: 7→216×10^9^/L) and significant tumor regression. Although disease progression occurred after 5 months, characterized by pulmonary metastasis, biliary obstruction and multiple infections, the substantial initial benefits of crizotinib cannot be overlooked. The patient survived 8 months from diagnosis, highlighting the transient efficacy of MET inhibition and the impact of clonal evolution. This case underscores the potential and limitations of MET inhibitors in gastric cancer. Biomarker-driven selection, early resistance detection, and trials exploring crizotinib-chemotherapy/immunotherapy combinations are urgently needed to improve outcomes in this aggressive subtype.

## 1 Introduction

Gastric cancer is a global health concern, often diagnosed at an advanced stage due to subtle early symptoms. In 2018, it accounted for 784,000 deaths, making it the third leading cause of cancer-related mortality worldwide (1). Among its subtypes, signet ring cell carcinoma (SRCC) is a highly malignant and poorly differentiated form of poorly cohesive carcinoma, characterized by diffuse infiltrative growth, high metastatic potential, and a late-stage diagnosis (2).

The prognosis of poorly cohesive carcinomas, including SRCC, remains controversial, largely due to variations in its definition. While some studies question whether SRCC has a worse prognosis compared to other gastric cancer types, its aggressive nature, chemoresistance, and high metastatic potential pose significant treatment challenges. Surgery is often only feasible in localized disease, and effective treatments for advanced SRCC remain limited. Molecularly targeted therapies, particularly those addressing oncogenic drivers, may provide new therapeutic avenues (3).

One emerging molecular target is the mesenchymal epithelial transition (MET) gene, which is located on the long arm of human chromosome 7. It encodes the cellular-mesenchymal epithelial transition (c-Met) protein, a receptor tyrosine kinase that normally regulates essential cellular processes including tumor proliferation, invasion, and poor prognosis in gastric cancer (4). However, MET amplification is relatively rare, occurring in only 2–10.2% of gastric cancers (5). MET protein overexpression is observed in approximately 50% of advanced cases (6), but only a subset of these patients exhibit true MET-driven oncogenesis, which is essential for response to targeted therapy. MET inhibitors like crizotinib have demonstrated significant clinical benefits in non-small cell lung cancer (NSCLC), particularly in patients with MET exon 14 skipping mutations(METΔ14) and high-level MET amplification (7). Early studies demonstrated that crizotinib has potential efficacy in MET-amplified gastric cancer (8). However, clinical outcomes have been inconsistent, likely due to tumor heterogeneity, clonal evolution, and microenvironmental influences. These factors highlight both the therapeutic potential of crizotinib and the need for further investigation to optimize its use in this patient population.

Here, we report a case of MET-amplified gastric cancer with systemic bone marrow metastases, a rare and aggressive disease presentation. The patient developed severe thrombocytopenia, a complication of disseminated carcinomatosis of the bone marrow (DCBM), and was treated with crizotinib. This case highlights the potential role of MET-targeted therapy in gastric cancer, while also underscoring the challenges of resistance and disease progression in this setting.

## 2 Case report

### 2.1 Patient information

This case report describes a 51-year-old female patient with no significant prior medical history. In July 2023, she presented with neck and upper back pain, along with left shoulder pain and restricted range of motion. She also reported occasional chest tightness, palpitations, fatigue, and scattered bruising on her limbs.

### 2.2 Clinical progression

**Figure 1:**
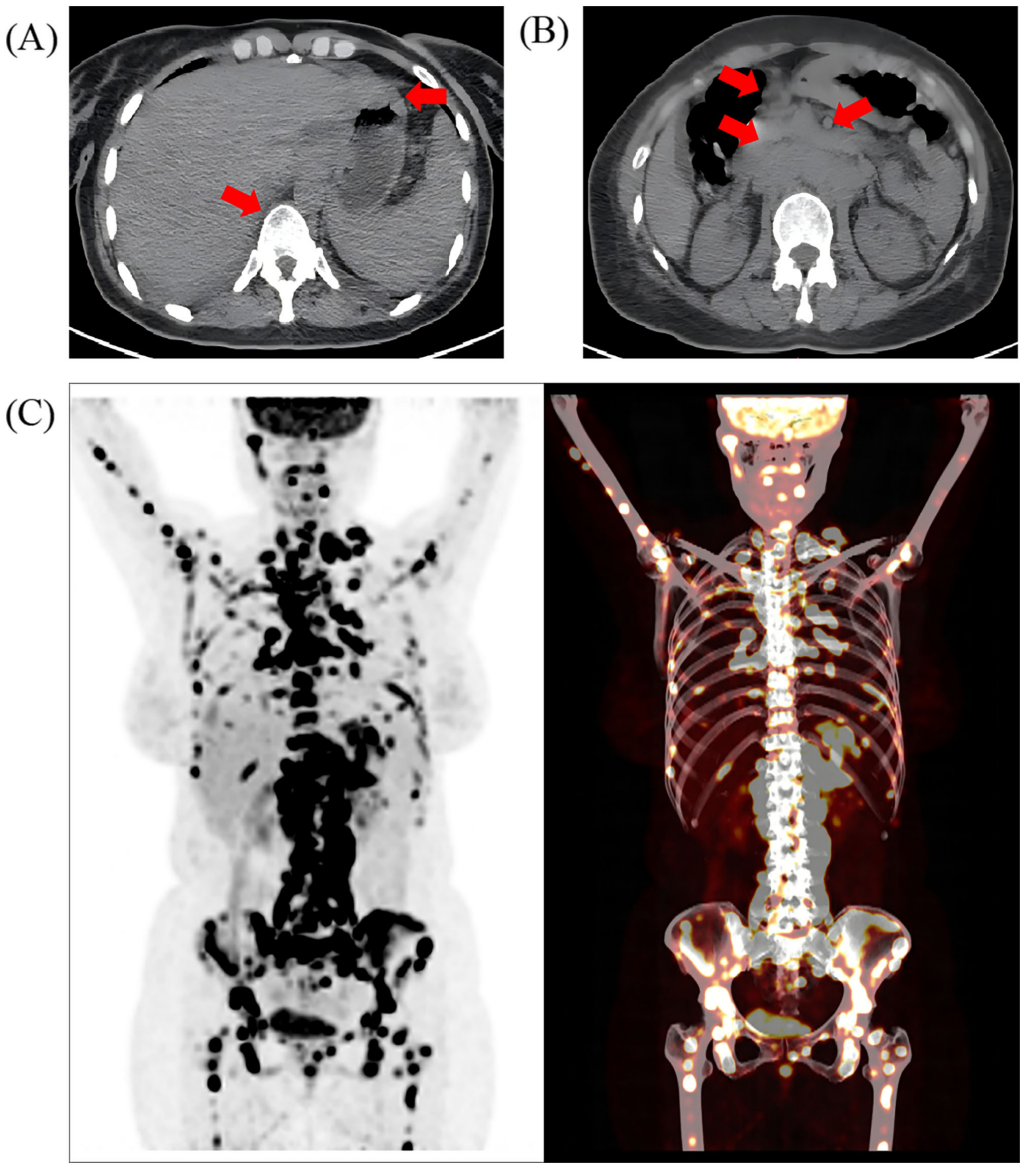
The imaging evaluation of the patient at admission. Positron emission computed tomography (PET-CT) showed **(A)** thickening of gastric wall as well as mixed metastasis of the vertebras and **(B)** multiple enlarged abdominal lymph nodes; the red arrow represents the location of the lesion; **(C)** increased FDG uptake in multiple areas of the bone marrow, including the skull, right mandible, several vertebrae and their attachments, sternum, multiple ribs, pelvic bones, clavicles, scapulae, humeri, and femurs.

Upon admission, laboratory tests revealed significantly elevated levels of carcinoembryonic antigen (CEA), CA153, CA125, CA724, and cytokeratin fragment 19. CT imaging indicated a suspected primary malignant pancreatic lesion with lymph node metastasis, raising concerns about both pancreatic malignancy and possible lymphoma. Additionally, diffuse thickening of the gastric wall was noted (**Figure 1A**), prompting recommendations for further endoscopic evaluation. PET-CT confirmed diffuse thickening of the gastric wall with irregularly increased Fluorodeoxyglucose (FDG) uptake, pancreatic swelling with diffuse FDG uptake, and multiple enlarged lymph nodes (**Figure 1B**) showing increased FDG metabolism in the bilateral cervical, supraclavicular and many other individual regions. Focal areas of increased FDG uptake were also observed throughout the bone marrow (**Figure 1C**), with several regions demonstrating osteolytic bone destruction.

These findings raised the possibility of a malignancy with multi-system involvement, particularly lymphoma, necessitating further pathological investigation. During the hospitalization, the patient developed progressive thrombocytopenia reaching a critically low platelet count of 7×10^9^/L, alongside elevated D-dimer levels, hypofibrinogenemia and prolonged prothrombin time. Persistent platelet transfusions and thrombopoietin therapy failed to restore platelet counts, with peak levels remaining subnormal at 39×10^9^/L. This indicated rapid progression of the malignancy with escalating tumor burden, placing the patient at significant bleeding risk and making it difficult to identify the primary lesion site.

### 2.3 Diagnosis and treatment

**Figure 2:**
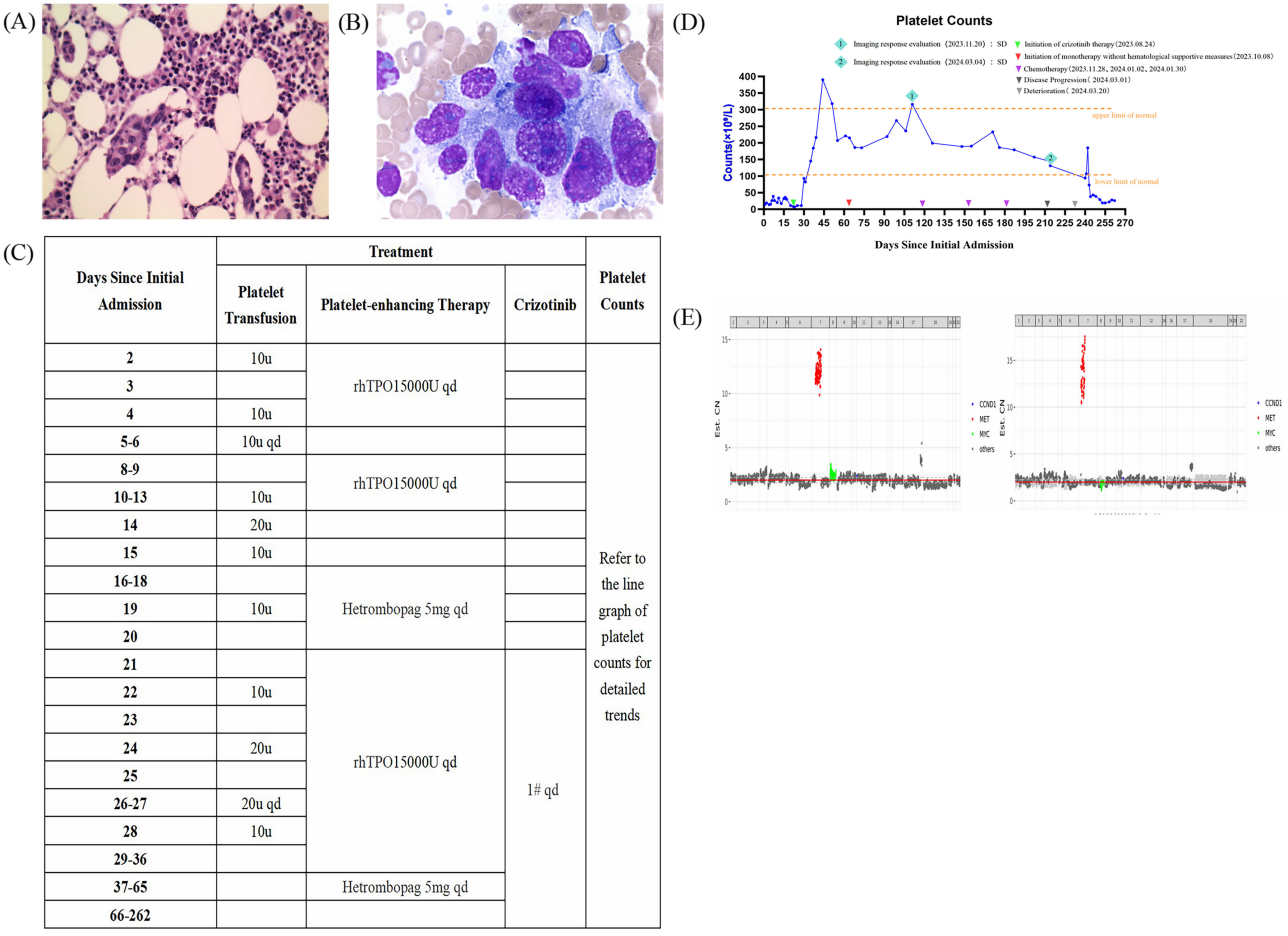
Bone marrow smear and biopsy of the patient after bone marrow infiltration and tendencies in hematopoietic function after the crizotinib treatments. Gene copy number distribution charts from the genetic reports. **(A)** The bone marrow smear and **(B)** the bone marrow biopsy showing metastatic poorly differentiated adenocarcinoma (hematoxylin-eosin, original magnification ×400). **(C)** Detailed supportive therapy and crizotinib administration timeline. **(D)** Dynamic monitoring of platelet counts suggested that the hematopoietic function was improved rapidly after the crizotinib treatments received in the hospital. E The gene copy number distribution charts obtained from the patient’s peripheral blood (left) and malignant bone marrow effusion (right).

The patient underwent a bone marrow biopsy (**Figure 2A**) and histological examination (**Figure 2B**), which ruled out lymphoma and confirmed metastatic poorly differentiated adenocarcinoma. Further investigation was recommended to locate the primary tumor, which was suspected to be in the stomach. Given the patient’s high bleeding risk from severe thrombocytopenia and coagulopathy, the oncology team suggested genetic testing. The next-generation sequencing (NGS) analysis of peripheral blood and malignant bone marrow effusion revealed focal amplifications of the MET gene on chromosome 7, with copy numbers exceeding 10 (**Figure 2E**). Moreover, no mutations were detected in other oncogenic drivers, including HER2, RAS/MAPK and PI3K/AKT pathways. Following multidisciplinary consultation with external specialists, the patient was initiated on crizotinib (1 tablet once daily). Remarkably, after treatment with this MET inhibitor, the patient’s hematopoietic function showed significant improvement. Her platelet count substantially increased to 93×10^9^/L within one week and normalized to 216×10^9^/L after two weeks of therapy (**Figures 2C, D**). Additionally, her coagulation function also improved rapidly.

### 2.4 Definitive diagnosis

On September 1, 2023, with the patient’s condition significantly improved, a gastroscopy was performed. The pathology results indicated poorly differentiated carcinoma at the angular incisure and low-adhesion carcinoma near the angular incisure in the gastric body, with areas of signet ring cell carcinoma. Lauren classification indicated a diffuse type. This led to the final diagnosis of gastric cancer.

### 2.5 Further treatment

**Figure 3:**
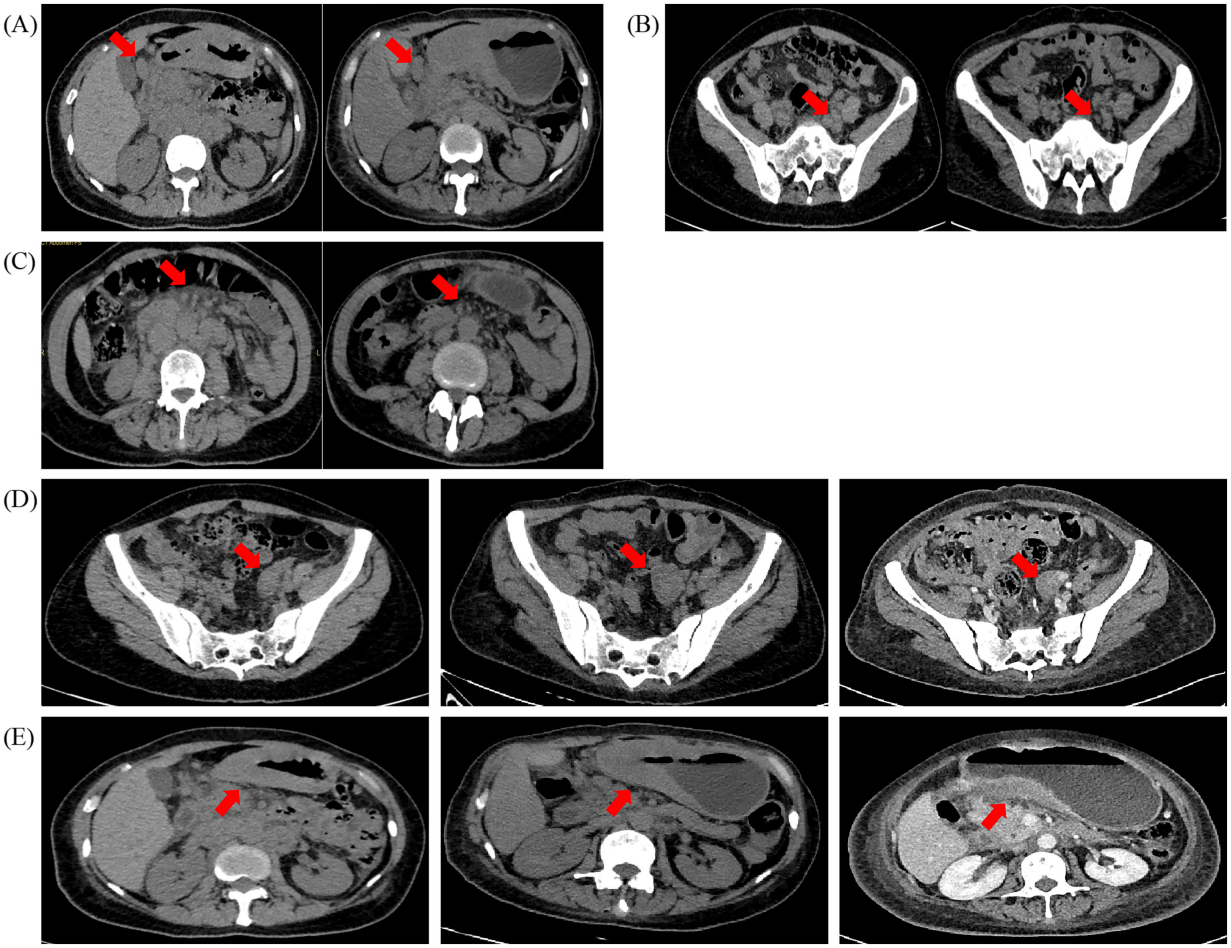
CT (Computed tomography) scans before (2023–08–03) and after (2023–11–20) (2024–03–04), the crizotinib treatments. **(A)** Metastatic lymph nodes around the gastric antrum and **(B)** pelvic metastatic lymph nodes were the two lesions that gradually shrunk significantly after the crizotinib treatments. **(C)** Omental metastatic lesions and **(D)** the pelvic adnexal mass shrunk significantly as well. E.The diffuse thickened gastric wall remained stable. All assessed lesions were indicated with red arrows.

After the treatment of crizotinib for three cycles, the patients’ subsequent imaging reports (**Figures 3A-E**) and laboratory test results showed marked improvement. According to RECIST 1.1 scoring standard, the therapeutic response was assessed as stable disease (SD), and the patient met the criteria for renewed chemotherapy eligibility. Consequently, the patient initiated a new anti-tumor regimen on November 28, 2023 for a total of three cycles, consisting of oxaliplatin combined with S-1 (Tegafur).

### 2.6 Disease progression

On March 1, 2024, the patient developed symptoms of cough, sputum production, and fatigue. The follow-up CT scan demonstrated increased bilateral pleural effusions and progressive omental lesions, while notably revealing significant size reduction of the pelvic adnexal mass (**Figure 3D**). Changes in some non-target lesions may be associated with inflammation or hypoalbuminemia and do not meet the criteria for progressive disease. According to RECIST 1.1 criteria, the therapeutic response after three cycles of chemotherapy was assessed as SD. Immunohistochemical analysis confirmed that the atypical cells observed in the pleural fluid were likely metastatic in origin from gastric carcinoma. Following this, the patient received relevant symptomatic and supportive treatments.

### 2.7 Deterioration

On March 20, 2024, the patient developed jaundice and abnormal liver function. It was considered that the intrahepatic bile duct dilatation, caused by internal liver compression, led to obstructive jaundice. On March 28, 2024, the patient underwent percutaneous transhepatic biliary drainage in the interventional radiology department. The patient developed polymicrobial infection after the surgery.

### 2.8 Outcome

**Figure 4:**
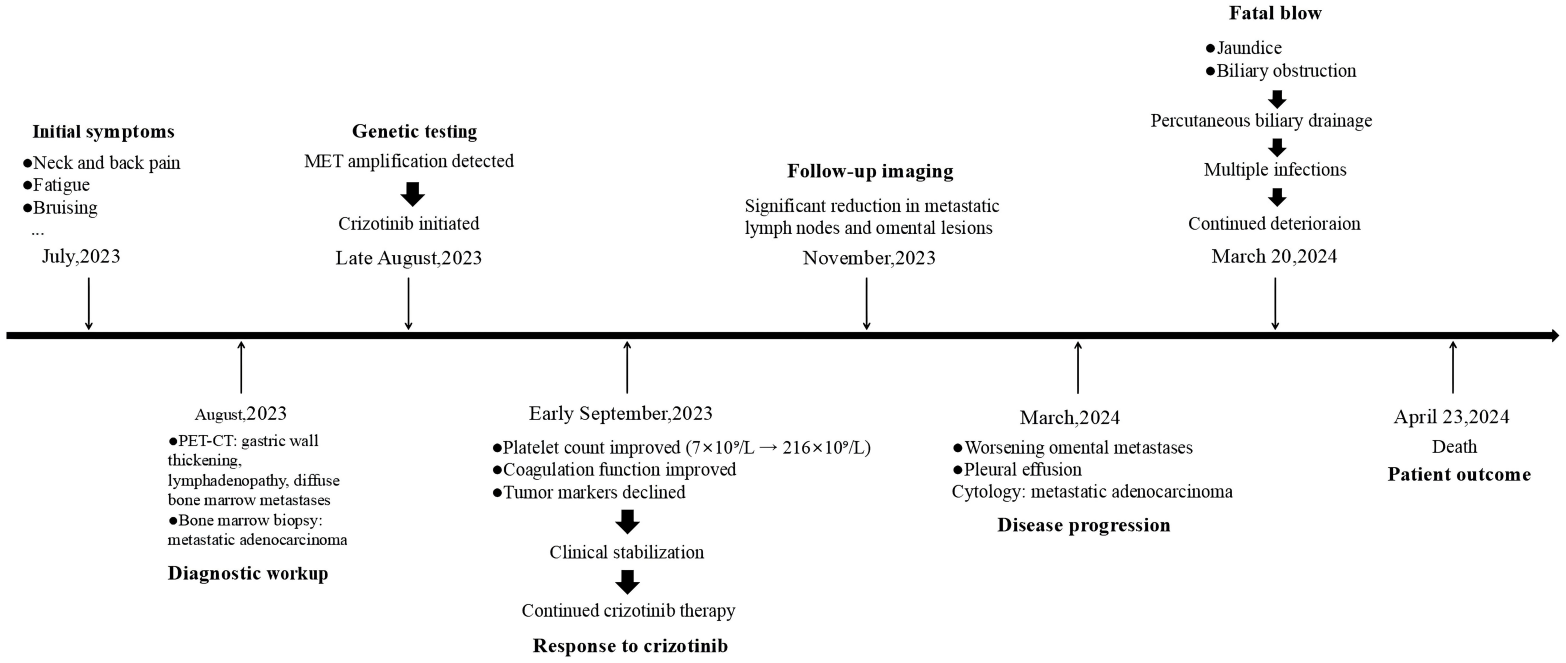
Timeline of the patient’s whole therapeutic process. Timeline for diagnosis and treatment form July 2023 to April 2024.

No alternative therapy initiated due to rapid clinical decline and family preference for palliative care, the patient was transferred back to a local hospital for treatment. On April 23, 2024, the patient was pronounced dead (**Figure 4**), with an overall survival of 8 months.

## 3 Discussion

Gastric adenocarcinoma accounts for approximately 95% of all gastric cancer cases and exhibits significant histological and molecular heterogeneity (9). The diffuse-type gastric cancer classified by the Lauren system, including signet ring cell carcinoma (SRCC), is characterized by poor differentiation, lack of cell adhesion, and an aggressive metastatic pattern, leading to a worse prognosis and limited treatment options (10) This patient’s disease, marked by systemic bone marrow metastasis, exemplifies the aggressive nature of this subtype, further compounded by a poor response to conventional therapies.

A significant challenge in this case was the development of severe thrombocytopenia and disseminated intravascular coagulation (DIC), conditions often associated with bone marrow metastasis. The concept of DCBM has been proposed in several case reports, referring to extensive bone marrow infiltration of solid tumors. DCBM is characterized by anemia, back pain, and bleeding tendencies, often associated with DIC, and can result in severe outcomes such as brain hemorrhage and death (11–20). Interestingly, in these reports, the most frequent primary tumors were either signet ring cell carcinoma or poorly cohesive carcinomas containing signet ring cell components (21). Infiltration of the bone marrow by highly aggressive tumor cells disrupts normal hematopoiesis and exacerbates pro-coagulant activity, leading to severe outcomes, as seen in this patient (22). This complex interaction between tumor burden and hematological complications underscores the need for early and aggressive management of the primary tumor in patients with widespread metastasis.

MET is involved in the metastatic progression of various cancers. In breast cancer, MET signaling activates the MAPK pathway and promotes the release of inflammatory cytokines such as IL-1β, IL-8, and CXCL1, thereby reshaping the brain microenvironment and enhancing brain metastasis (23). In lung cancer, METΔ14 leads to sustained MET activation, enhancing cell migration and resistance to apoptosis (24);TP53 - Induced Glycolysis and Apoptosis Regulator (TIGAR) has also been reported to promote lung cancer cell migration and metastasis via MET (25). In hepatocellular carcinoma, aberrant activation of the HGF/MET axis promotes angiogenesis and is closely associated with metastasis and drug resistance (26). Increased MET expression in metastatic cells of head and neck cancer is considered a marker of lymph node metastasis (27). In certain renal cancer stem cells, MET is overexpressed and contributes to bone metastasis (28). MET amplification is a well-established driver of tumor proliferation, invasion, and metastasis (29), particularly in poorly cohesive gastric carcinomas. A study has indicated that in gastric cancer, MET amplification drives epithelial–mesenchymal transition (EMT), extracellular matrix degradation via MMPs, and VEGF pathway activation, collectively promoting metastasis (30). Cross-tumor evidence supports MET as a key factor in the formation of metastatic niches. Its active role in the rare but fatal bone marrow metastasis observed in this case highlights the therapeutic potential of MET inhibitors, such as crizotinib, in metastatic gastric cancer. However, the heterogeneous response—where some lesions shrank while others progressed—highlights the challenges of treating a genetically diverse tumor.

A key challenge in MET-targeted therapy lies in the correlation between treatment efficacy and MET amplification levels. Current evidence suggests that patients with high-level MET amplification respond better to MET inhibitors (31–35), yet standardized thresholds remain elusive. The 2023 NCCN guidelines define high amplification as MET copy number >10 via NGS (36), while FISH categorizes amplification based on MET/Centromere of Chromosome 7 (CEP7) ratios (low: 1.8–2.2; intermediate: >2.2–<5; high: ≥5) (37). In our case, NGS revealed focal MET amplification (copy number >10), a subtype validated as a clinically significant driver. However, NGS has notable limitations (1): it cannot assess CEP7 status, and (2) its algorithms for copy number calculation (based on sequencing depth and variant allele frequency) require refinement (38). Studies indicate that even with high tumor content (≥10%) and deep sequencing (≥500×), concordance between NGS and FISH for MET amplification is only 62.5%, and NGS results poorly correlate with clinical outcomes, underscoring the need for multi-platform validation (39). Establishing robust biomarker-based screening methods could help identify patients most likely to benefit from MET-targeted therapies and optimize treatment strategies (40, 41). Furthermore, although *in vitro* and *in vivo* studies have shown that gastric cancer cell lines are highly sensitive to crizotinib (8, 41–43), large-scale clinical trials in gastric cancer patients are lacking. This limits the ability to draw definitive conclusions about its efficacy and broad applicability in treating MET-amplified gastric cancers. Additionally, baseline genetic testing was conducted at diagnosis. However, repeat testing was not performed in later treatment stages despite declining efficacy of targeted therapy. This gap hinders identification of secondary resistance mechanisms, such as clonal evolution and therapeutic selection pressure which may arise during treatment.

The heterogeneous treatment response observed in this patient may reflect clonal evolution within the tumor. Different metastatic sites may harbor distinct genetic mutations and resistance mechanisms (44–46), leading to variable drug sensitivity. Under selective pressure from targeted therapy, tumor cells with pre-existing or acquired resistance mutations may become the dominant clones, reducing treatment efficacy (47). Some studies have suggested that high-level MET amplification (≥5 gene copies per cell) correlates with greater sensitivity to MET inhibitors, whereas lower amplification levels or concurrent resistance mutations (e.g., RAS or PI3K pathway alterations) may limit benefit (35, 48). Although the initial pre-treatment genetic testing did not detect any relevant alterations, clonal evolution and therapeutic selection pressure may lead to divergent genomic profiles, including altered amplification status, emerging resistance mutations, or activation of bypass pathways. Unfortunately, follow-up testing was not conducted. Additionally, our initial gene panel did not include Programmed cell death ligand 1(PD-L1) expression status, limiting comprehensive assessment of its therapeutic benefit. Beyond intrinsic genetic factors, the tumor microenvironment (TME) plays a crucial role in modulating drug response, as certain sites could have been more conducive to tumor growth or more resistant to treatment (44). Differences in stromal support, angiogenesis, and immune cell infiltration, among other factors, may lead to varying sensitivity to MET inhibition across different metastatic sites (47). Studies in NSCLC suggest that MET-amplified tumors often exhibit immune exclusion, which could further diminish responses to targeted therapy (49).Given the heterogeneity and clonal evolution of MET-amplified tumors, monotherapy with MET inhibitors like crizotinib may be insufficient for durable disease control. Combination therapy strategies are being actively explored to overcome resistance and enhance efficacy: Combining MET inhibitors with chemotherapy (e.g., fluorouracil, leucovorin, and oxaliplatin in the mFOLFOX6 regimen) has shown synergistic effects in some studies, as chemotherapy may help target resistant tumor subclones while maintaining selective MET inhibition (50).MET amplification is often associated with an immunosuppressive tumor microenvironment, suggesting a potential role for immune checkpoint inhibitors (e.g., anti-PD-1/PD-L1 therapy) in combination with MET-targeted therapy. In NSCLC, early trials combining MET inhibitors with immune checkpoint blockade have yielded promising results, and similar strategies could be evaluated in gastric cancer (49). Other approaches, such as simultaneous inhibition of MET and alternative oncogenic pathways (e.g., PI3K, EGFR, or VEGF signaling), may further enhance treatment efficacy and delay resistance (51).

These therapeutic challenges were acutely evident in our patient’s case. Despite the potential of combination strategies, clinical urgency necessitated a prioritized intervention. This case report outlines the clinical trajectory of a single patient, and its observational design inherently precludes causal conclusions. The absence of a control cohort (e.g., MET-amplified patients managed with standard chemotherapy or best supportive care) limits direct comparisons between crizotinib and alternative therapies. Nevertheless, the patient’s rapid clinical decline—characterized by severe thrombocytopenia (7×10^9^/L) and disseminated intravascular coagulation—posed an immediate threat, rendering conventional chemotherapy (e.g., fluorouracil-platinum combinations) exceptionally high-risk. We feared chemotherapy’s myelosuppressive effects might worsen blood toxicity, potentially triggering life-threatening bleeding or infections. In contrast, crizotinib, an oral MET inhibitor, demonstrated a favorable hematologic safety profile in prior studies, alongside preliminary evidence of antitumor activity in MET-amplified malignancies (8, 43). Following multidisciplinary consensus, crizotinib was selected as the initial therapy to swiftly reduce tumor burden, stabilize blood counts, and establish a foundation for subsequent chemotherapy. While these observations highlight the potential utility of MET inhibition in this critical context, the lack of controlled data underscores the urgent need for prospective trials or matched cohort analyses to validate the relative efficacy of MET-targeted strategies in similar high-risk populations.

This case highlights the critical role of molecular profiling in guiding targeted therapy. The identification of MET amplification facilitated the use of crizotinib, which led to rapid improvement in thrombocytopenia and coagulation abnormalities. However, the emergence of resistance and subsequent disease progression emphasize the necessity of (1): Comprehensive genomic profiling (e.g., NGS, FISH, and liquid biopsy) to enable real-time detection of resistant clones (2). Multi-region tumor sampling to account for intratumoral heterogeneity and identify potential resistance mechanisms (3). Longitudinal molecular monitoring to inform adaptive treatment strategies and facilitate timely therapeutic adjustments. Future clinical practice should integrate routine molecular profiling to enable truly personalized therapeutic interventions.

## 4 Conclusion

In summary, this case highlights the potential of crizotinib in MET-amplified gastric cancer while also illustrating the challenges posed by tumor heterogeneity and acquired resistance. The mixed response observed in this patient suggests that biomarker-driven selection and combination strategies may be necessary to optimize MET-targeted therapy. This case further reinforces the importance of molecular profiling in guiding treatment decisions and underscores the urgent need for expanded clinical research on MET inhibitors in gastric cancer.

## Data Availability

The original contributions presented in the study are included in the article/supplementary material. Further inquiries can be directed to the corresponding author.
